# Impact of hospital characteristics on implementation of a Pediatric Early Warning System in resource-limited cancer hospitals

**DOI:** 10.3389/fonc.2023.1122355

**Published:** 2023-05-03

**Authors:** Farris Abutineh, Dylan E. Graetz, Hilmarie Muniz-Talavera, Gia Ferrara, Maria Puerto-Torres, Yichen Chen, Srinithya R. Gillipelli, Paul Elish, Alejandra Gonzalez-Ruiz, Yvania Alfonso Carreras, Shillel Alvarez, Daniela Arce Cabrera, Deiby Arguello Vargas, Miriam Armenta Cruz, Camila Barra, Patricia Calderon Sotelo, Zulma Carpio, Mayra Chavez Rios, Daniela Covarrubias, Lucy de Leon Vasquez, Rosdali Diaz Coronado, Ever Amilcar Fing Soto, Wendy Gomez-Garcia, Cinthia Hernandez, María Susana Juarez Tobias, Esmeralda Leon, Jose de Jesus Loeza Oliva, Alejandra Mendez, Kenia Miller, Erika Montalvo Cozar, Natalia del Carmen Negroe Ocampo, Eulalia Penafiel, Estuardo Pineda, Ligia Rios, Esperanza Rodriguez Ordonez, Veronica Soto Chavez, Meenakshi Devidas, Asya Agulnik

**Affiliations:** ^1^ Department of Global Pediatric Medicine, St. Jude Children’s Research Hospital, Memphis, TN, United States; ^2^ Baylor College of Medicine, Houston, TX, United States; ^3^ Rollins School of Public Health, Emory University, Atlanta, GA, United States; ^4^ Abt Associates, Rockville, MD, United States; ^5^ Pediatric Oncology, Hospital Saint-Damien, Port-au-Prince, Haiti; ^6^ Pediatric Oncology, Benemérito Hospital General con Especialidades “Juan María de Salvatierr”, La Paz, Mexico; ^7^ Pediatric Hemato-Oncology Unit, Hospital Pediatrico de Sinaloa, Culiacan, Mexico; ^8^ Hemato-Oncology, Hospital Nacional de Niños, San Jose, Costa Rica; ^9^ Pediatric Oncology, Hospital General de Tijuana, Tijuana, Mexico; ^10^ Pediatric Oncology, Centro de Investigacion Bradford Hill, Santiago, Chile; ^11^ Pediatric Oncology, Hospital Infantil Manuel Rivera La Mascota, Managua, Nicaragua; ^12^ Pediatric Oncology, Instituto Nacional de Enfermedades Neoplasticas, Lima, Peru; ^13^ Hemato-Oncology, Hospital para el Niño Poblano, Puebla, Mexico; ^14^ Pediatric Oncology, Centro Estatal de Oncología de Campeche, Campeche, Mexico; ^15^ Pediatric Oncology, Hospital Infantil Regional Universitario Dr. Arturo Grullon, Santiago, Dominican Republic; ^16^ Pediatric Oncology, Hospital General Celaya, Celaya, Mexico; ^17^ Oncology Unit, Hospital Infantil Dr. Robert Reid Cabral, Santo, Domingo, Dominican Republic; ^18^ Nursing, Hospital Infantil Teletón de Oncología, Queretaro, Mexico; ^19^ Pediatric, Hospital Central Dr. Ignacio Morones Prieto, San Luis Potosi, Mexico; ^20^ Medical Oncology, Hospital Guillermo Almenara Irigoyen, Lima, Peru; ^21^ Pediatric Oncology, Centro Estatal de Cancerologia, Xalapa, Mexico; ^22^ Pediatric Hemato-Oncology, Hospital del Niño “Jose Renan Esquivel”, Panama City, Panama; ^23^ Pediatric Intensive Care Unit, Hospital Oncológico Solca Núcleo de Quito, Quito, Ecuador; ^24^ Pediatric Hemato-Oncology, Hospital General Agustin O’Horan, Merida, Mexico; ^25^ Pediatric Oncology, Instituto del Cáncer SOLCA Cuenca, Cuenca, Ecuador; ^26^ Pediatric Hemato-Oncology, Hospital Nacional de Niños Benjamín Bloom, San Salvador, El Salvador; ^27^ Pediatric Hemato-Oncology, Hospital Nacional Edgardo Rebagliati Martins, Lima, Peru; ^28^ Pediatric Oncology, Centro Medico Nacional Siglo XXI, Mexico City, Mexico; ^29^ Pediatric Hemato-Oncology, Hospital Civil de Guadalajara, Guadalajara, Mexico

**Keywords:** Pediatric Early Warning Systems (PEWS), quality improvement collaborative (QIC), implementation science, pediatric oncology, resource-limited settings, global health

## Abstract

**Background:**

Pediatric Early Warning Systems (PEWS) aid in identification of deterioration in hospitalized children with cancer but are underutilized in resource-limited settings. Proyecto EVAT is a multicenter quality improvement (QI) collaborative in Latin America to implement PEWS. This study investigates the relationship between hospital characteristics and time required for PEWS implementation.

**Methods:**

This convergent mixed-methods study included 23 Proyecto EVAT childhood cancer centers; 5 hospitals representing quick and slow implementers were selected for qualitative analysis. Semi-structured interviews were conducted with 71 stakeholders involved in PEWS implementation. Interviews were recorded, transcribed and translated to English, then coded using *a priori* and novel codes. Thematic content analysis explored the impact of *hospital characteristics* and *QI experience* on time required for PEWS implementation and was supplemented by quantitative analysis exploring the relationship between hospital characteristics and implementation time.

**Results:**

In both quantitative and qualitative analysis, material and human resources to support PEWS significantly impacted time to implementation. Lack of resources produced various obstacles that extended time necessary for centers to achieve successful implementation. Hospital characteristics, such as funding structure and type, influenced PEWS implementation time by determining their resource-availability. Prior hospital or implementation leader experience with QI, however, helped facilitate implementation by assisting implementers predict and overcome resource-related challenges.

**Conclusions:**

Hospital characteristics impact time required to implement PEWS in resource-limited childhood cancer centers; however, prior QI experience helps anticipate and adapt to resource challenges and more quickly implement PEWS. QI training should be a component of strategies to scale-up use of evidence-based interventions like PEWS in resource-limited settings.

## Introduction

With modern advancements in treatments and supportive care, survival of children with cancer in high-income countries has risen to over 80% (1, 2). However, survival in low-middle-income countries (LMICs), where roughly 90% of children with cancer reside (1), remains low, between 10% and 50% (1, 3). Treatment-related toxicity (3) and infections (4) contribute to cancer mortality in resource-limited settings, where hospitals face limitations in staff and equipment needed for supportive care (5–10). There is an urgent need for evidence-based practices that reduce preventable mortality and improve global childhood cancer survival.

Pediatric Early Warning Systems (PEWS) are evidence-based interventions that allow for early detection of clinical deterioration in hospitalized children with cancer (11–13). PEWS produce multi-level advantages beyond the patient (14), such as improving interdisciplinary (15) and family communication (16), reducing hospital costs (17), and empowering providers (18). Resource-limited hospitals, however, face additional challenges implementing PEWS (19). More work is needed to understand how to address implementation challenges and support PEWS adoption in these settings.

The Consolidated Framework for Implementation Research (CFIR) describes factors influencing implementation of evidence-based interventions across five domains: *inner setting, characteristics of individuals, outer setting, intervention characteristics, and implementation process* (20–22), with modifications suggested for LMICs (22). CFIR constructs like *culture* (23), *individual need* (23), and *teaming* (23) characterize different aspects of the implementation process and their impact on its outcomes, e.g. time (24). The inner setting domain, including characteristics like resource availability and infrastructure, has been identified as particularly relevant to implementation of evidence-based interventions in resource-limited hospitals (22, 25, 26). Our prior work similarly suggested the importance of hospital characteristics on PEWS implementation (5); however, it remains unclear *how* these characteristics influence time required to implement PEWS, or what strategies can mitigate these effects. In this study, we evaluate the impact of hospital characteristics on PEWS implementation time in resource-limited pediatric oncology centers.

## Methods

### Setting

Proyecto Escala de Valoración de Alerta Temprana (EVAT) is a multicenter quality improvement (QI) collaborative in Latin America to implement PEWS (12). At participating centers, local implementation teams work with regional PEWS experts to plan, pilot, implement, and assess impact of PEWS (5, 27).

### Data collection

This mixed-methods study included 23 Proyecto EVAT centers across 11 Latin American countries completing PEWS implementation prior to March 2020. Time required for PEWS implementation was calculated from the start of the PEWS pilot to implementation completion.

Qualitative data collection has been described previously (5). Briefly, we selected 5 centers representing extremes of implementation time for in-depth analysis, including 3 high-performing centers (3-4 months for PEWS implementation) and 2 low-performing centers (10-11 months). At each center, two researchers conducted semi-structured interviews with 10 to 15 stakeholders involved in PEWS implementation, including hospital directors, PEWS implementation leaders, or other staff (see **Supplementary Table 1** for participant demographics). Interviews were conducted virtually using WebEx, recorded, transcribed, and translated to English for analysis.

Quantitative data included measures of various center features. Initially collected on enrollment in Proyecto EVAT, site leads confirmed hospital data at the start of this study.

### Definitions

Consistent with Proyecto EVAT criteria, “implementation completion” was defined as having at least 2 months with high-quality PEWS use (5, 27). Centers are considered to have high-quality PEWS use when they have less than 15% in the three types of PEWS use errors: errors in PEWS scoring, PEWS algorithm non-adherence, and PEWS omissions (documented vital signs without using PEWS) (27). Implementation time was defined as time from the PEWS pilot start to implementation completion.

For analysis, research team members *a priori* identified hospital attributes hypothesized to be related to PEWS implementation time; these were supplemented with data from quantitative findings during analysis. Their definitions can also be found in **Supplementary Table 2**.

Hospital *material resources* included pediatric intensive care unit (PICU) capacity, physical pediatric hematology-oncology (PHO) ward space, and available finances. PICU capacity described available space in the ICU where pediatric patients were treated or the total number of PICU beds. Physical space was described by the number of beds per shared room on the PHO ward. Available finances describe available hospital economic resources for equipment and supplies needed for PEWS.

*Human resources* included the PHO ward nurse-to-patient ratio, number of PICU physicians, and staff turnover (how often hospital staff are replaced by new staff). In quantitative analysis, the nurse-to-patient ratio was interpreted according to the International Society of Paediatric Oncology (SIOP) nursing standards for LMICs, which recommend a ratio of one nurse to five or fewer pediatric oncology patients (28, 29). The number of PICU physicians included pediatric intensivists, fellows, and other critical care providers with expertise treating critically ill children with cancer.

*Hospital characteristics* encompassed funding structure (public or private), type (academic or not, specialized or general), relative PHO patient prioritization, and PHO service complexity. Specialized hospitals consisted of oncology or pediatric multidisciplinary centers while general hospitals included both general and women children’s hospitals. PHO patient prioritization conveyed the relative importance placed on PHO patient care and was quantitatively described by number of PHO beds and PHO ward structure (separate PHO ward or general pediatric ward). Service complexity was measured by the number of wards requiring PEWS implementation and number of staff requiring PEWS training.

Finally, we characterized hospitals by the participants’ self-reported prior individual or institutional *experience with QI* initiatives.

### Data analysis

This study used a convergent mixed method design to investigate hospital characteristics that impact PEWS implementation time. For qualitative data, the study team developed a codebook *a priori* from the CFIR (20, 21) and supplemented by novel codes from iterative transcript review. Two researchers coded transcripts using the 2020 edition of MAXQDA software (VERBI Software GmbH), achieving a kappa of 0.8 to 0.9.

We used thematic content analysis focusing on the impact of *hospital characteristics* and *QI experience* on time required for PEWS implementation (**Supplementary Table 3** for code definitions) Constant comparative analysis was used to explore perceived characteristics related to PEWS implementation across different hospitals and participant roles.

Quantitative analyses evaluated the relationship between hospital characteristics and PEWS implementation time. Association of PEWS implementation time with categorical and continuous covariates were analyzed using Wilcoxon rank sum test and univariate non-parametric regression analysis (Theil-Sen median estimators), respectively. P-values < 0.05 were considered statistically significant. Analyses were conducted using R 4.2.0 (https://www.r-project.org/).

We iteratively compared quantitative and qualitative results to synthesize common themes and statistical trends of how hospital characteristics related to PEWS implementation time.

## Results

**Figure 1:**
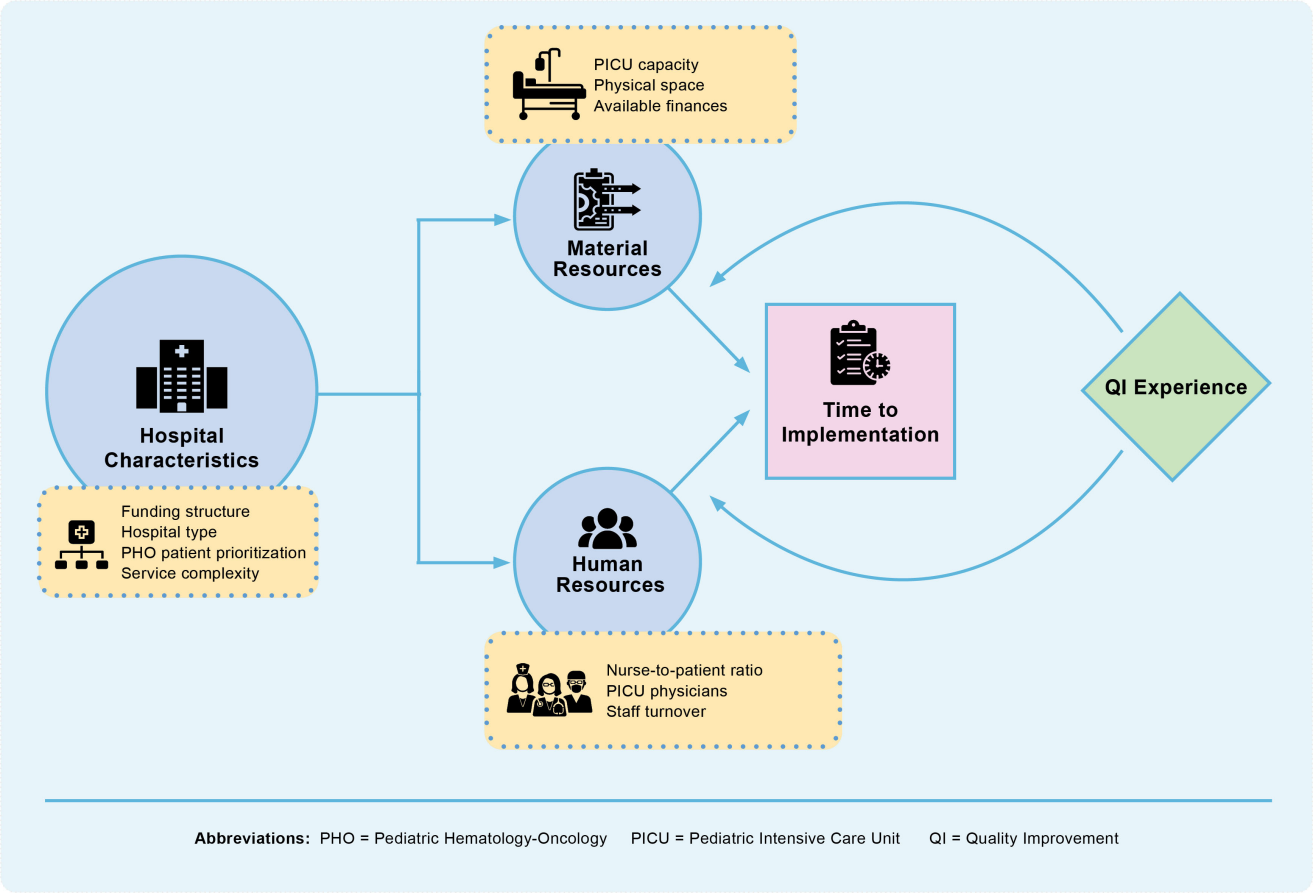
During implementation of Pediatric Early Warning Systems (PEWS), hospital characteristics such as funding structure and type impact resource availability, which in turn influences time required to implement PEWS. A hospital’s experience with Quality Improvement (QI) can alter this relationship between its resource availability and time to implementation, supporting faster PEWS implementation by enabling implementers to proactively identify and address PEWS barriers.

Mixed methods analysis identified multiple factors associated with time required to implement PEWS, including material and human resources, hospital characteristics, and QI experience (**Figure 1**).

### Material resources

In qualitative analysis, participants described various material resource limitations that impacted time for PEWS implementation, including PICU capacity, physical space, and available finances (**Table 1**).

**Table 1:** Participant perspectives on material and human resources.

Theme	Sub-theme	Example Quote
Material Resources	PICU capacity	“because my hospital doesn’t have intensive care for children, we have limitations, so, finally we end up treating children who should be in ICU on the service floor until we can get a bed in ICU” (nurse director, Lima)
		“One of the biggest limitations has been the number of patients and beds, and the deficit in beds is more notable” (physician director, Lima)
	Physical space	“So our demand from oncology patients is very high. For example, we used to have 8 beds and we would reach up to 372 oncology admission, just to our service” (nurse director, SLP)
		“a little girl had just died of a common situation, a patient who was not assisted in the general room, died and she could have been saved” (implementation leader, Xalapa)
	Available finances	“we didn’t have bracelets of every size to measure the blood pressure, we didn’t have oximeters, the stethoscopes we used were bad quality, some old, damaged, they even hurt the ears” (implementation leader, Xalapa)
		“We committed, in the training, to the acquisition of equipment so the staff could do it. Because if they had the training but not the equipment or the supplies it wouldn’t work” (nurse director, El Salvador)
Human Resources	Nurse-to-patient ratio	“our department has 22 beds, we are a very small team of nurses and I think that was the main obstacle and we thought that was not going allow us to implement in our department” (implementation leader, Cuenca)
		“Among the barriers we found, there was the human resources, we didn’t have … we used to be 1 nurse for 8 patients” (implementation leader, Lima)
	PICU physicians	“The other limitation we already talked about is that we don’t have an intensivist for every shift … the ideal thing would be to have an intensivist who can evaluate the patient because I think they have more experience and can better manage the deterioration” (physician director, SLP)
		“the limitations we have are staff … we had to be careful to select what patients receive the intervention because if we did the intervention to all patients then probably our team would have been insufficient” (physician director, Lima)
	Staff turnover	“We have a high level of absenteeism in the hospital, the ones that stayed are ready to a leave, so every time we’re less people working “(implementation leader, Xalapa)
		“we have people taking leaves, human resource is very limited … the absenteeism, the load of work, is a huge barrier for us” (implementation leader, El Salvador)

Participants at hospitals with limited PICU capacity had challenges implementing PEWS due to limited ability to transfer a patient with deterioration to a higher level-of-care: *“There are few beds in the [P]ICU, so when the patient needed to be transferred because he was getting worse, there was no free space*” (physician director, San Louis Potosi [SLP]). In hospitals without a dedicated PICU, pediatric patients were admitted to adult ICUs, further stretching limited resources: “*it’s a multi-use ICU … we had to manage bed limitation to admit both adult patients and pediatric patients … we don’t have the necessary number of beds to treat all patients*” (physician director, Lima).

Similarly, hospitals’ physical space limitation obstructed implementation of PEWS: “*reduced space where we cannot monitor the child 24/7…made it difficult to find the way to the patient and move him to a space for higher supervision*” (implementation leader, SLP). Additionally, financial limitations increased time required for PEWS implementation as hospitals struggled to obtain necessary medical equipment: “*I wanted to do things well, but I didn’t have the equipment, and I ended up doing nothing*” (implementation leader, Xalapa).

Quantitative data supported these findings (**Table 2**); hospitals with more PICU beds required less time for PEWS implementation (p = 0.045) and those with fewer beds per shared room implemented faster (p = < 0.0001).

**Table 2:** Association of continuous data with implementation time.

Characteristic	Min	Median	Max	p*
Number of PICU beds	0	8	27	0.045
Number of beds per shared room	1	4	15	<0.0001
Number of PICU physicians	0	3	28	0.18
Number of PHO beds	0	22	65	0.039
Number of staff (physicians + nurses) requiring PEWS training	16	49	901	0.0014

PICU, pediatric intensive care unit; PHO, pediatric hematology-oncology; PEWS, pediatric early warning systems.

p*: p-values using univariate nonparametric regression analyses (Theil-Sen single median estimator).

### Human resources

In qualitative analysis, participants also identified human resource limitations that impacted PEWS implementation time, including nurse-to-patient ratios, availability of PICU physicians, and staff turnover (**Table 1**).

A low ratio of clinical staff to patient volume increased workload and threatened the quality of patient care, including ability to use PEWS. Nurses especially voiced this concern: “*we’ve tried to have one nurse per child … taking care of one child implies a bigger effort and that couldn’t be shared if there was an extra adult or child*” (nurse director, Lima). Nurses across all hospitals considered high nurse-to-patient ratios a significant barrier to PEWS use: “*we have a big workload, one nurse for 8 or 9 patients, sometimes 11…the human factor is a big barrier for us … we cannot manage that”* (implementation leader, El Salvador).

Similarly, hospitals lacking physicians specialized in PICU management struggled with timely evaluation and transfer of deteriorating patients, negatively affecting both patient outcomes and PEWS implementation: “*We only work with one on-call intensivist … when they call saying this patient is having a cardiac arrest, even though I’d do everything in my power, I won’t be able to get there in time*” (implementation leader, Cuenca). Even in settings with adequate physician staffing, a lack of specialists trained in management of critically ill children with cancer was felt to increase implementation time: “*pediatrics is not our chosen specialty … Even though we have all the knowledge and experience from the courses, the health care staff don’t have the vocation or the affinity to work with children*” (physician director, Lima).

In some hospitals, staff turnover, through both absenteeism and rotations, prolonged implementation as it was necessary to retrain staff in PEWS, and new staff without prior training struggled to consistently use PEWS correctly: “*the new [resident] comes in … without good training, so some things may happen regarding the management that are incorrect*” (physician director, SLP).

This perceived relationship between human resources and time to PEWS implementation was not observed in the quantitative analysis; neither the number of PICU physicians, nor the nurse-to-patient ratio significantly impacted implementation time (p = 0.18, **Table 2** and p = 0.85, **Table 3**, respectively). The relationship between nurse-to-patient ratio and implementation time can be visualized with **Supplementary Figure 1**.

**Table 3:** Association of categorical data with implementation time.

Characteristic		n	%	t (median months)	p**
**Nurse-to-patient ratio (1 nurse to how many patients)**					**0.85**
	Five or less	9	39	6.0	
	Greater than five	14	61	6.0	
**Funding structure**					**0.94**
	Public	18	78	6.0	
	Private + Mix (public/private)	5	22	5.5	
**Hospital type**					NA*
	Academic	22	96	6.0	
	Non-academic	1	4	8.4	
	General (general + women children’s hospital)	9	39	7.0	**0.025**
	Specialized (pediatric multidisciplinary + oncology)	14	61	5.2	
**PHO ward structure**					**0.071**
	Separate PHO ward	21	91	6.0	
	No PHO ward (general pediatric only)	2	9	9.7	
**Number of PHO wards requiring PEWS implementation**					**0.013**
	One ward	19	83	5.5	
	More than one ward	4	17	9.6	
**QI Experience**					**0.13**
	Yes	6	26	4.5	
	No	17	74	6.5	

PHO, pediatric hematology-oncology; PEWS, pediatric early warning systems; QI, quality improvement.

p**: p-value using Wilcoxon rank sum test.

NA*: Analysis unavailable for academic hospital type due to low sample size (1 non-academic hospital).

### Hospital characteristics

Across all hospitals, hospital characteristics such as funding structure, type, and PHO patient prioritization were seen by participants to impact PEWS implementation time by determining the relative availability of material and human resources for PEWS (**Table 4**).

**Table 4:** Participant perspectives on hospital characteristics and QI experience.

Theme	Sub-theme	Example Quote
Hospital Characteristics	Funding structure	“we’re a public hospital and we have limited economic resources” (implementation leader, Xalapa)
		“one thing that I see as important is this hospital is not a public hospital that depends on state resources, because probably things are slower” (research director, Cuenca)
	Academic centers	“the non-academic would be the fastest, the academic hospitals would the slowest because they prepare human resources” (quality director, SLP)
		We’re an academic hospital … they come to our hospital to become pediatricians, surgeon” (QI coordinator, El Salvador).
	Hospital type	“general hospitals don’t offer pediatric oncology services because they don’t have enough specialists and they don’t have enough technology” (physician director, Lima)
		“it’s a general hospital. In that service we treat from onco-hematology patients to surgery patients, so we don’t only treat oncology patients, maybe 60%” (nurse director, SLP)
QI Experience	Impact on implementation	“like I was telling you we have 10 years working on continuous quality improvement programs, in the oncology service, the nurses already had their equipment and they gave orientation seminars to all the staff and training” (QI coordinator, El Salvador)
		“because we had traveled some part of the road already … we had to follow certain standards for attention, so, when PEWS came we had all this background and it was easier to make it run” (data manager, Xalapa)
	Plans for future initiatives	“This was the example to have better or bigger projects in quality improvement in order to help us with the rest of the processes at the hospital” (implementation leader, Lima).
		“we proposed that PEWS could be implemented to other departments, general pediatrics, pulmonology, etc. not only oncology” (physician director, El Salvador).

PICU, pediatric intensive care unit; QI, quality improvement.

Public hospitals rely on government resources, and participants from these settings described their centers as frequently underfunded, reducing available material and human resources necessary to quickly implement PEWS: “*Our country is a poor country, our hospital is a public hospital, we lack many resources and it’s difficult to request them*” (implementation leader, Lima). Conversely, participants viewed private hospitals as having greater access to human and material resources and fewer administrative barriers when requesting resources for new projects: “*our hospital is a hospital that has its own resources. We were able to quickly approve it and prove that this was a sustainable project which helped the implementation go faster*” (research director, Cuenca).

Similarly, academic teaching hospitals were perceived as having less resources and thus required more time for PEWS adoption. Academic hospitals faced more implementation barriers due to the prioritization of training healthcare staff, thus reducing time for initiatives like PEWS: *“[Non-academic hospitals] can dedicate all the time to assisting patients. In academic hospitals, you have the excuse of preparing human resources, so it’s not feasible to develop certain types of initiatives*” (quality director, SLP). In some teaching facilities, trainees with limited experience managing pediatric emergencies increased implementation time: *“We don’t prepare residents in pediatric emergencies … so the hospitals that prepare residents in pediatric intensive care would have an earlier adoption than us”* (quality director, SLP). Additionally, academic hospitals experienced more rotations among trainees, contributing to issues with PEWS use: *“[in an academic hospital] they complete their training period, and they leave … So, it’s very variable to capture the critical state of a patient”* (physician director, SLP).

Finally, participants across all centers reported that specialized hospitals, such as pediatric multidisciplinary or oncology hospitals, encountered fewer implementation barriers due to staff experience with and institutional prioritization of pediatric and/or oncology patients: “*Since we are an oncology hospital … we try to be updated and have good reception for those programs that strengthen our patient’s safety*” (nurse director, Xalapa). General hospitals were felt to have other competing priorities and less experience with pediatric oncology, resulting in fewer resources for projects like PEWS: “*This generated some rejection because our [general] hospital has limited resources and we would need oximeters for children*” (implementation leader, Lima).

Of the 23 participating hospitals, only 2 were private and 3 were mixed private/public; in quantitative analysis, we did not find an association between funding structure and implementation time (p = 0.94, **Table 3**). Similarly, only 1 hospital was non-academic, preventing analysis of the relationship between academic status and implementation time. Aligned with qualitative findings, however, quantitative analysis demonstrated that specialized hospitals implemented faster than general hospitals (p = 0.025, **Table 3**). Hospital prioritization of PHO patients was also significantly related to PEWS implementation time; hospitals with more PHO inpatient beds implemented faster (p = 0.039, **Table 2**), and those with a dedicated PHO ward trended towards shorter implementation times (p = 0.071, **Table 3**).

In quantitative analysis, service complexity emerged as an additional barrier to PEWS implementation. Hospitals with more than one PHO ward requiring PEWS implementation and those with more nurses and physicians requiring PEWS training required more time for PEWS implementation (p = 0.013, **Table 3** and p = 0.0014, **Table 2**, respectively). Further conceptualization of various hospital characteristics impact on implementation time are available in **Supplementary Figures 2A–D** respectively.

### QI experience

Prior QI experience, both at the hospital and among implementation team members, was seen by participants to facilitate PEWS implementation by allowing centers to more easily overcome existing resource limitations (**Table 4**). Examples of these experiences included involvement with initiatives related to central venous catheters, decreasing hospitalization times, and shortening time to antibiotic administration in febrile neutropenia.

Past experience with QI was seen to facilitate PEWS implementation by allowing centers to anticipate and proactively address potential implementation barriers: “*I think the knowledge exchange allows you to identify the difficulties you have in your center and learn from the experience of other centers*” (physician director, Lima). Nurses also felt empowered by QI experience to participate in PEWS implementation as members of the multidisciplinary team: “*since I’m a nurse I know how to take care of a patient, that [and to learn about quality] facilitated my support to conducting that project [PEWS] and to my colleagues*” (nurse director, El Salvador).

Conversely, hospitals without QI experience struggled with implementation and were initially intimidated by the PEWS project: “*it was something big … maybe we wouldn’t be able to accomplish it … maybe most of us felt the same way about not being able to accomplish it*” (implementation leader, Cuenca). Despite most hospitals lacking prior QI experience, all eventually achieved successful PEWS implementation, often applying QI methodology learned in Proyecto EVAT: *“At the beginning, it was kind of a barrier because we were afraid of the unknown, but then we were very successful*” (implementation leader, Cuenca). Successfully implementing PEWS also empowered hospitals to apply their experience to future improvement initiatives: “*A lot of us have started to get involved in other quality improvement projects that maybe didn’t exist before PEWS, it has helped us and pushed us to work*” (implementation leader, Lima).

Most hospitals lacked QI experience prior to PEWS implementation (n = 17, 74%, **Table 3**). Supporting qualitative findings, quantitative analysis demonstrated hospitals without QI experience trended towards longer PEWS implementation times (6.5 months vs. 4.5 months, p = 0.13, **Table 3**). This relationship is also displayed in **Supplementary Figure 3**.

## Discussion

This study analyzed the relationship between hospital characteristics and PEWS implementation time in resource-limited settings. Fixed hospital characteristics, like funding structure and type, determined the relative availability of resources for PEWS and impacted time needed for implementation. Previous QI experience, however, either at the center or among members of the implementation team, mitigated these barriers by empowering centers to proactively anticipate and overcome implementation challenges. In centers without prior QI experience, implementation leaders leveraged training obtained through Proyecto EVAT to successfully implement PEWS.

Our findings are consistent with prior work in LMICs demonstrating the impact of resource availability on QI and intervention implementation (7, 8), including the barriers of staff turnover (26, 30), large organization size (31), and poor infrastructure (25). Similarly, the importance of hospital and staff specialization have been identified as important to the quality and capacity of pediatric onco-critical care (10, 32). Additionally, a systematic review evaluating the use of the CFIR in LMICs proposed a new domain, “Characteristics of Systems,” that affects organizational policies to produce changes to the inner setting (hospital) domain (22). This relationship reflects the impact of hospital characteristics (e.g., funding structure) on resource-availability we observed in this study.

Although data on the impact of QI collaboratives in LMICs is conflicting (33), our work supports findings that including QI training, as is done in Proyecto EVAT, can improve collaborative effectiveness (33, 34). In this study, few centers or implementation team members reported previous experience with QI, highlighting the importance of incorporating QI training into programs to scale-up interventions in resource-limited hospitals. Our findings suggest that QI training also provides additional benefits, including team empowerment and motivation to introduce other improvement projects, potentially resulting in more broad impact on patient outcomes.

Centers in our study more quickly completed implementation when they adapted the PEWS implementation process to the specific characteristics of their institution and resource-level. These findings provide actionable recommendations for clinicians, hospital leadership, and researchers wishing to implement PEWS or other QI interventions in resource-limited clinical settings. For clinicians, we recommend an iterative implementation strategy that includes aspects of successful methodologies from other resource-constrained sites and tailoring them to the needs of their center. This can include formal QI methods such as plan-do-study-act (PDSA) cycles, stakeholder analyses, and process mapping, among others. Hospital leadership looking to foster a culture of QI in their hospital should support local QI efforts and promote QI training options within the center to grow institutional and clinician capacity for QI. For researchers and public health experts leading collaborative efforts to scale-up evidence-based interventions, we recommend including training in QI methodology to better enable clinicians to leverage their knowledge to support improvement initiatives.

This study has several limitations. The relatively small sample size (23 centers) and low frequency of some variables (e.g., private funding structure) limited the power of our quantitative analysis to identify true relationships between some variables and PEWS implementation time. Our mixed methods design, however, supplemented this quantitative data with in-depth qualitative analysis from a diverse group of stakeholders. The synthesis between quantitative and qualitative findings strengthened our study and enriched the analysis of the relationship between hospital characteristics and PEWS implementation. At the time of this study, all Proyecto EVAT centers had successfully implemented PEWS (27). As a result, we used time needed for implementation, rather than implementation success or failure, as the implementation outcome. Implementation time is a relatively newly described implementation outcome (24), and this study further contributes to this emerging literature. Finally, this study focused on implementation of one intervention in pediatric oncology centers, potentially limiting generalizability of our findings to other interventions and settings. Future work should more broadly evaluate the impact of hospital characteristics on implementation of other interventions to improve childhood cancer care and explore the impact of external factors (e.g., the COVID pandemic) on intervention implementation and sustainability in resource-limited settings (35). This includes evaluation of the impact of changes in resources to promote intervention use over time and study of associated intervention costs and cost-benefits and their impact on sustainability.

## Conclusions

This study describes how hospital characteristics impact time required for successful PEWS implementation in resource-limited pediatric oncology centers, with past hospital or individual QI experience mitigating implementation challenges by empowering implementation teams to proactively overcome identified barriers. Importantly, lack of prior QI experience can be addressed through teaching QI methods as part of the implementation process. These findings can be used by clinicians and researchers to conduct pre-implementation assessments to anticipate implementation challenges and guide future collaborative initiatives to scale up interventions that improve outcomes of children with cancer in hospitals of all resource-levels.

## Data availability statement

The raw data supporting the conclusions of this article will be made available by the authors, without undue reservation.

## Ethics statement

This study was approved by the institutional review board of St. Jude Children’s Research Hospital as an exempt, minimal-risk study. Additional approvals were obtained by participating centers as needed. As an exempt study, written participant consent was waived; verbal consent was provided at the start of each interview.

## Author contributions

AA, DG developed the idea. MP-T, SG, PE, HM-T, AG-R, MA, CB, ZC, CH, MJ, JJL, AM, EM, EPe, EPi collected the data. AA provided supervision. FA and AA conducted the data analyses. FA, AA, DG drafted manuscript and prepared the tables and figures. All authors contributed to the interpretation of the findings, the editing of the article, and the approval of the final submitted version.
