# Supplementary material for: Impact of hospital characteristics on implementation of a Pediatric Early Warning System in resource-limited cancer hospitals
Source: Front Oncol. 2023 May 3;13:1122355. doi: 10.3389/fonc.2023.1122355 (PMC10189109; doi:10.3389/fonc.2023.1122355)
Supplement: Supplementary file 1 [file DataSheet_1.docx]

***Supplementary Material***

**Impact of Hospital Characteristics on Implementation of a Pediatric Early Warning System (PEWS) in Resource-Limited Cancer Hospitals**

**Farris Abutineh, BS^1^; Dylan E. Graetz, MD, MPH^1^; Hilmarie Muniz-Talavera, PhD^1^; Gia Ferrara, MSGH^1^; Maria Puerto-Torres, BA^1^; Yichen Chen^1^; Srinithya R Gillipelli, BA^2^; Paul Elish, MPH^3^; Alejandra Gonzalez-Ruiz, MD, MIH^4^; Yvania Alfonso Carreras, MD^5^; Shillel Alvarez, RN^6^; Daniela Arce Cabrera, MD^7^; Deiby Arguello Vargas, RN^8^; Miriam Armenta Cruz, RN^9^; Camila Barra, RN^10^; Patricia Calderon Sotelo, MD^11^; Zulma Carpio, RN^12^; Mayra Chavez Rios, MD^13^; Daniela Covarrubias, MD^14^; Lucy de Leon Vasquez, MD^15^; Rosdali Diaz Coronado, MD^12^; Ever Amilcar Fing Soto, RN^16^; Wendy Gomez-Garcia, MD^17^; Cinthia Hernandez, RN^18^; María Susana Juarez Tobias, MD^19^; Esmeralda Leon, MD^20^; Jose de Jesus Loeza Oliva, MD^21^; Alejandra Mendez, MD^1^; Kenia Miller, MD^22^; Erika Montalvo Cozar, MD^23^; Natalia del Carmen Negroe Ocampo, MD^24^; Eulalia Penafiel, MD^25^; Estuardo Pineda, MD^26^; Ligia Rios, MD^27^; Esperanza Rodriguez Ordonez, MD^28^; Veronica Soto Chavez, MD^29^; Meenakshi Devidas, PhD^1^; Asya Agulnik, MD, MPH^1*^**

***Correspondence:**

Asya Agulnik

asya.agulnik@stjude.org

**Supplementary Table 1**: **Participant Demographics***

| **Characteristic** | **n (%)** |
| --- | --- |
| **Center** | |
| Lima, Peru | 18 (25.4) |
| San Luis Potosi, Mexico | 11 (15.5) |
| San Salvador, El Salvador | 15 (21.1) |
| Cuenca, Ecuador | 15 (21.1) |
| Xalapa, Mexico | 12 (16.9) |
|  | |
| **Profession** | |
| Floor Physician | 26 (36.6) |
| ICU Physician | 6 (8.5) |
| Nurse | 32 (45.1) |
| Other | 7 (9.9) |
|  | |
| **Sex** | |
| Male | 21 (29.6) |
| Female | 50 (70.4) |
|  | |
| **Years working in center** | |
| 0 – 10 | 27 (38.0) |
| 11-20 | 25 (35.2) |
| ≥21 | 19 (26.8) |
|  | |
| **Role in Center** | |
| Administrator | 8 (11.3) |
| Clinician | 30 (42.3) |
| Clinician-director | 33 (46.5) |
|  | |
| **Role in PEWS Implementation** | |
| Implementation leader | 39 (54.9) |
| Director | 21 (29.6) |
| Other | 11 (15.5) |

**Abbreviations**: ICU – Intensive Care Unit, PEWS – Pediatric Early Warning Systems

*adapted from Agulnik et al. *Assessment of Barriers and Enablers to Implementation of a Pediatric Early Warning System in Resource-Limited Settings.*

**Supplementary Table 2: Definitions**

| **Theme** | **Sub-theme** | **Analysis** | **Definition** |
| --- | --- | --- | --- |
| Material Resources | PICU capacity | Qual | Describes the available space in the ICU where pediatric patients were treated relative to patient volume |
|  |  | Quant | Number of PICU beds |
|  | Physical space | Qual | Amount of available space for staff to implement and use PEWS |
|  |  | Quant | Number of beds per shared room |
|  | Available finances | Qual | Institute’s capacity for economic contribution to the equipment and bed requirements for PEWS implementation and use |
| Human Resources | Nurse-to-patient ratio | Qual | The number of patients each nurse implementing PEWS was responsible for |
|  |  | Quant | Nurse-to-patient ratio |
|  | PICU physicians | Qual | Number of, specialization, and comfort of physicians to treat critically ill children with cancer, includes all pediatric intensivists, fellows, and critical care providers |
|  |  | Quant | Number of PICU physicians |
|  | Staff turnover | Qual | The number of or rate at which old staff are replaced by new staff |
| Hospital Characteristics | Funding structure | Qual | Whether the hospital obtained its funding through public (government-assisted) or private means |
|  |  | Quant | Public vs. Private + Mix (private/public) |
|  | Hospital type | Qual | Whether the hospital specialized in treating pediatric, oncology, or both patients or was generalized; also addresses whether or not it was an academic training center |
|  |  | Quant | Specialized (pediatric multidisciplinary + oncology) vs. General (general + women children’s hospital) |
|  | PHO patient prioritization | Qual | Level of importance/priority hospitals place on care of PHO patients |
|  |  | Quant | Number of PHO beds |
|  |  |  | PHO ward structure (separate ward or integrated into another) |
|  | Service complexity | Quant | Number of PHO wards requiring PEWS implementation (1 vs. more than 1) |
|  |  |  | Number of staff (nurses + physicians) requiring PEWS training |
| QI Experience | Impact on implementation | Qual | How participants/hospitals’ past QI experience affected their ability to implement PEWS |
|  |  | Quant | Did they (participant/hospital) have QI experience (Yes vs. No) |
|  | Plans for future initiatives | Qual | How participants/hospitals leverage their newly gained QI experience for future projects in the hospital, both PEWS related and non-PEWS related |

**Abbreviations**: PICU = pediatric intensive care unit, PHO = pediatric hematology/oncology, QI-Quality Improvement; PEWS-Pediatric Early Warning Systems

**Supplementary Table 3: Code Book**

| **Category** | **Code** | **Definition** |
| --- | --- | --- |
| Inner Setting | Hospital Characteristics | Any description of the hospital including organization, type of funding, size, age, etc. Includes description of the pediatric hematology-oncology unit vs other units, general mentions of staffing and workload (if not related to PEWS). Does not include mention of organizations culture (code as "Culture"). |
|  | QI Experience | Experience of the hospital or individuals in quality improvement projects in any setting prior to PEWS, includes lack of experience. |
|  | Available Material Resources | The level of resources dedicated to implementation and ongoing use of PEWS, including money, physical space, and materials. This includes monitors and other vital sign equipment as well as PEWS guides or tools, patient boards. Includes mention of how resources were obtained, if mentioning specific resources delegated for this. If the reference is unclear for material vs human resources, code both. |
|  | Available Human Resources | The level of human resources dedicated to implementation and ongoing use of PEWS, including personnel and time, or having enough time dedicated for PEWS training (including providing paid time to attend trainings). If the reference is unclear for material vs human resources, code both. |
|  | Staff Turnover | Any mention of staff turnover, including ration nurses, residents, fellows, physicians, or entry of new staff to the unit. Also includes turn-over of leadership in the hospital. Includes mentions of lack of turnover (staff permanence). If mention of training new staff, double code with "PEWS Training". |
|  | Why PEWS | Why the hospital decided to implement PEWS, including the reason behind the perceived need for this intervention at the center, or how the current situation is intolerable. Frequently involves statements such as "before PEWS (something bad happened to patients)" or "we needed PEWS to improve... in our hospital". Do not double code with "Goals". |
|  | Culture | The approach (norms, values, and basic assumptions) of personnel in the hospital to patient safety and quality, including the desire to do what's best for the patient and provide high-quality patient-centered care, and other elements of the hospital's general culture. Includes mentions employees feeling valued, essential, and psychologically safe to try new methods, including how new ideas for improvements are approached and adopted and how leadership generally respond to new suggestions for improvements. Includes general mentions of how decisions are made and hierarchies. |
|  | Site Adaptation | The degree to which PEWS aligns or does not align with the existing values and structure of the hospital. This includes mention of changes made in the institution to be able to implement PEWS (such as increased staffing, changing nursing flowsheets, changing the frequency of doing vital signs), as well as mentions of the compatibility, or 'fit' between PEWS and the hospital's existing workflows and systems or the values of individuals who work there. This does not include changes made to the PEWS too, algorithm, or system (code that as "Adaptability OF PEWS"). |
|  | Role of Leadership/Authorities | Commitment, involvement, and accountability of leaders and managers with implementation and use of PEWS, including their role in PEWS. Includes statements like "the leadership was supportive" and giving mandates to do XYZ with PEWS and general awareness about PEWS. |
|  | Outside Help | Individuals or organizations from OUTSIDE the institution who formally influenced or helped implement PEWS. Includes mention of people coming from other centers to train staff, going to other centers for training, experience with HOW other centers implemented PEWS. Includes communication or support given to the center by outside individuals during implementation. |

**Supplementary Figure 1: Implementation time by Nurse-to-Patient Ratio**


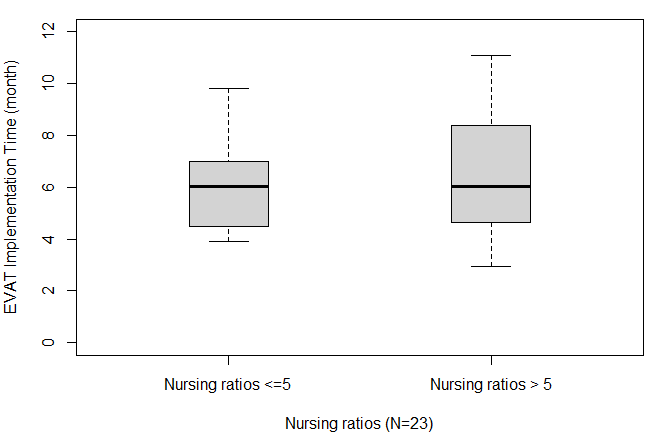


**Supplementary Figure 1.** Demonstrates the relationship between PEWS implementation time and hospital nurse-to-patient ratio.

Supplementary Figure 2a: Implementation Time by Funding Structure


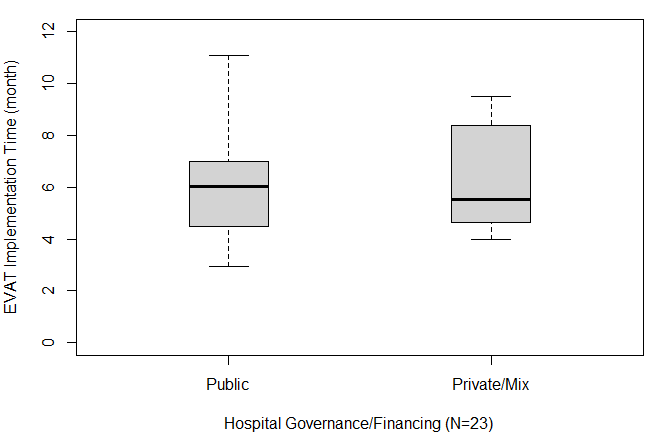


**Supplementary Figure 2a.** Demonstrates the relationship between PEWS implementation time and hospital funding structure.

Supplementary Figure 2b: Implementation Time by Hospital Type


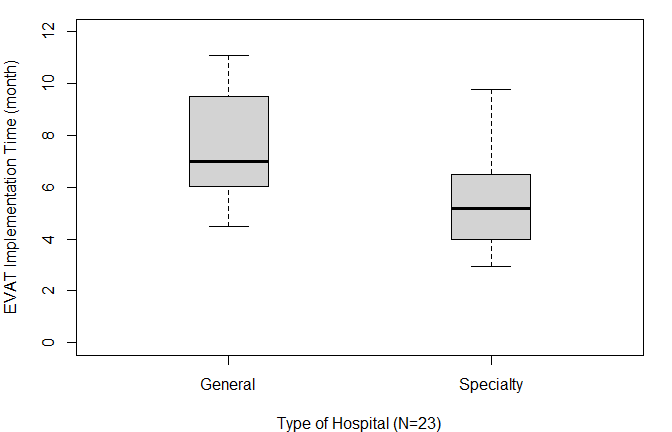


**Supplementary Figure 2b.** Demonstrates the relationship between PEWS implementation time and hospital type.

Supplementary Figure 2c: Implementation Time by PHO Ward Structure


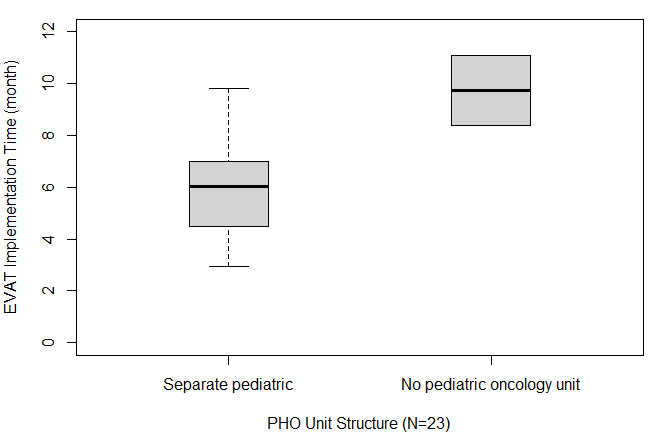


**Supplementary Figure 2c.** Demonstrates the relationship between PEWS implementation time and hospital PHO ward structure

Supplementary Figure 2d: Implementation Time by Number of PHO Wards Requiring PEWS Implementation


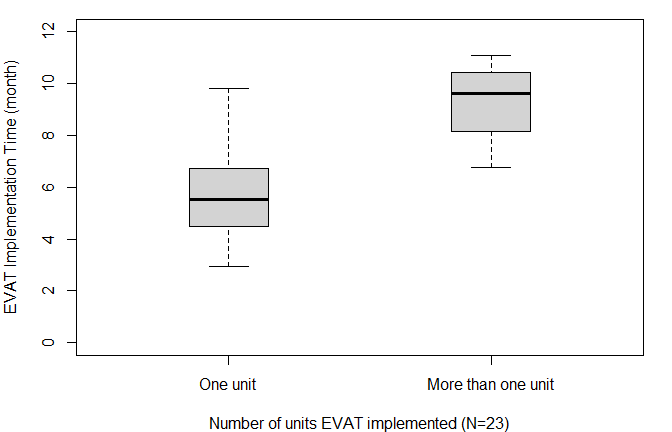


**Supplementary Figure 2d.** Demonstrates the relationship between PEWS implementation time and hospital number of PHO wards requiring PEWS implementation.

Supplementary Figure 3: Implementation Time by Hospital or Implementation Leader Past QI Experience


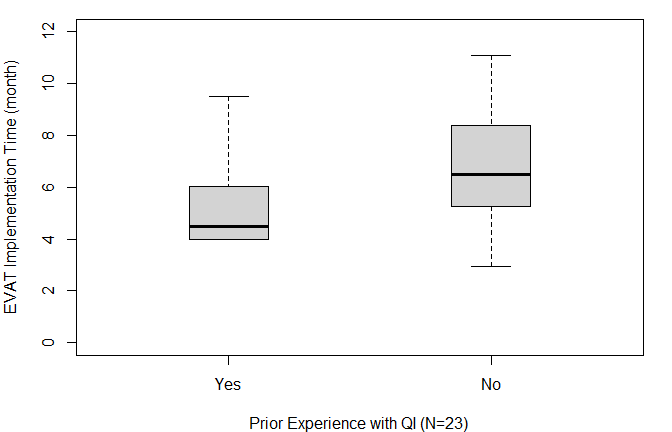


**Supplementary Figure 3.** Demonstrates the relationship between PEWS implementation time and hospital or implementer experience with quality improvement.
